# Disc prolapse and cord contusion in a case of Klippel-Feil syndrome following minor trauma

**DOI:** 10.4103/0019-5413.50857

**Published:** 2009

**Authors:** Amit Agrawal, Arvind M Badve, Nikhil Swarnkar, Kaustubh Sarda

**Affiliations:** Department of Surgery, Datta Meghe Institute of Medical Sciences, Sawangi (Meghe), Wardha, India; 1 Department of Anesthesiology, Datta Meghe Institute of Medical Sciences, Sawangi (Meghe), Wardha, India

**Keywords:** Cervical disc, Klippel-Feil anomaly, trauma

## Abstract

Klippel-Feil syndrome (KFS) is defined as congenital fusion of two or more cervical vertebrae and patients with KFS are frequently asymptomatic. However, these patients are especially prone to cervical cord injury after a minor fall or a major traumatic episode. We report an unusual case of KFS where the patient had disc prolapse between two Klippel-Feil segments and discuss the difficulties in the management of this case.

## INTRODUCTION

Klippel-Feil syndrome (KFS) is defined as congenital fusion of two or more cervical vertebrae and results from faulty segmentation of the vertebral axis during weeks 3-8 of gestation.^1^,^2^ Although patients with KFS are frequently asymptomatic, these patients are especially prone to cervical cord injury after a minor fall or a major traumatic episode.^2^–^4^ We report an unusual case of KFS where the patient had disc prolapse between two Klippel-Feil segments and discuss the difficulties in the management of this case.

## CASE REPORT

**Figure 1:**
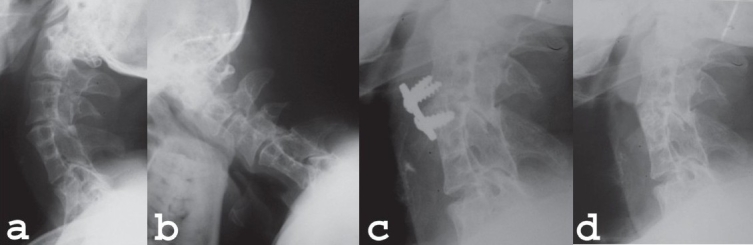
Lateral x-rays of cervical spine (a) showing C3-4 and C5-7 vertebral body fusion with waist formation. The lower surface of C4 is concave and upper surface of C5 in convex. Lateral X-ray cervical spine (b) in flexion shows movements of C4 over C5 vertebral body. (c) Postoperative lateral radiograph of the same patient shows C4-5 interbody plates and screws fixation with screw pulling out. (d) Follow-up X-ray after implant removal shows evidence of sound body fusion

**Figure 2:**
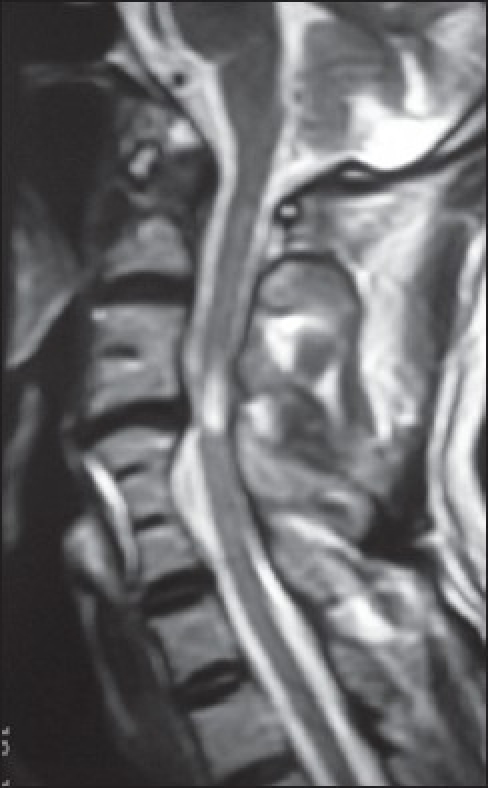
T2-weighted magnetic resonance image showing a large C4-5 disc prolapse resulting in compression of the dural tube and hyperintensity in the adjoining spinal cord

A 40-year-old man presented with complaints of weakness, tingling and numbness in all four limbs following fall from bicycle. Following the fall he had quadriparesis with muscle power grade 4+/5. The deep tendon jerks were exaggerated in all four limbs. He used to walk with support. The bowel and bladder functions were normal. The posterior column sensations were moderately impaired in the lower limbs. Bilateral plantars were extensor. The gag and palate reflexes were normal. X-ray films of the cervical spine showed fusion of cervical vertebrae at two levels i.e. the C3 and C4 vertebrae and C5, 6 and C7 vertebrae with spinal instability [Figure 1a and 1b]. Magnetic resonance imaging (MRI) of the cervical spine showed congenital fusion of the same vertebral bodies and in addition it also showed disc prolapse at C4-5 level with cord contusion at the same level [Figure 2]. The patient underwent right anterior cervical approach, C4-5 discectomy, fusion with autologus bone graft and C3-C4 fixation with titanium plate and screws [Figure 1c]. Following surgery hard cervical collar was applied and all activities related to neck movements were restricted for a period of six weeks. The patient made gradual recovery. At four months follow-up the power in all four limbs was grade 4+/5 and he was able to walk without support and had full control of bowel and bladder. The posterior column sensation recovered. However, because of screw pull out we had to remove the implants once there was evidence of bony fusion [Figure 1d].

## DISCUSSION

**Figure 3:**
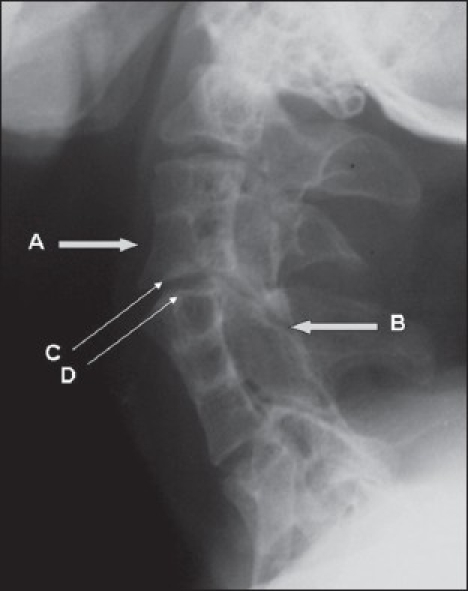
Preoperative X-ray (lateral view) cervical spine shows altered transfer of mechanical force to adjacent hypermobile segments (arrows A and B) that was further aggravated by concavo-convex joint surface (arrows C and D)

Spondylotic and discogenic changes occur in the junctional segments in association with cervical instability resulting from hypermobile segments adjacent to the fused vertebrae. A symptomatic herniated cervical disc may develop from an excessive mechanical load stress in KFS patients with multiple fused segments.^3^,^5^ As in the present case patients with KFS may be at an increased risk of neurological injury as a result of hypermobility of the various cervical segments. This predisposition to spinal cord injury has been attributed to the fused segments and the resultant altered mechanical force transfer that makes the adjacent nonfused segments excessively mobile [Figure 3].^2^–^4^ The cervical spine is unable to compensate for excessive flexion, extension, rotation and lateral bending. The block vertebrae transfer forces through long mechanical lever arms to the adjacent hypermobile segments. The more the levels fused, the more patients are predisposed to excess motion and overloading at the remaining mobile segment which leads to accelerated discogenic degenerative disease with bulging or herniated disks with subsequent risk of post-traumatic neurological sequelae.^4^,^6^ Treatment regimens depend on the severity of symptomatic segmental instability or neurological compromise, varying from modification of activities to extensive spinal surgery.^7^ As described we opted for microsurgical removal of the herniated disc via an anterior approach that was followed by interbody fixation with anterior plating.^5^,^8^ However, in the present case short neck and distorted vertebral anatomy posed a difficult challenge both in the exposure of the spine and in the placement of plate. In KFS cases arthroplasty with the Bryan artificial disc has been reported as the most suitable choice in young patients with the aim to preserve the motion in the remaining cervical spine segments which is important for maintaining neck function and quality of life.^9^,^10^ However, this was not feasible because of high cost in our case. As in the present case, persons with Klippel-Feil syndrome may be at increased risk of sustaining a neurological deficit even after minor trauma and appropriate guidance should be provided to alter their behavior to avoid any episode of neurological compromise.^6^
